# Genome-Wide Identification and Expression Analysis of the Histone Deacetylase Gene Family in Wheat (*Triticum aestivum* L.)

**DOI:** 10.3390/plants10010019

**Published:** 2020-12-24

**Authors:** Peng Jin, Shiqi Gao, Long He, Miaoze Xu, Tianye Zhang, Fan Zhang, Yaoyao Jiang, Tingting Liu, Jin Yang, Jian Yang, Liangying Dai, Jianping Chen

**Affiliations:** 1 College of Plant Protection, Hunan Agricultural University, Changsha 410128, China; PengJ0310@163.com; 2 State Key Laboratory for Quality and Safety of Agro-Products, Institute of Plant Virology, Ningbo University, Ningbo 315211, China; SqGao1997@163.com (S.G.); hnndhelong2@163.com (L.H.); xumiaoze@yeah.net (M.X.); ZTye1995@163.com (T.Z.); zf950418@163.com (F.Z.); 18868192310@163.com (Y.J.); anatkh6@163.com (T.L.); yjsamily@163.com (J.Y.); yangjian@nbu.edu.cn (J.Y.)

**Keywords:** wheat, histone deacetylase (HDAC), genome-wide, expression pattern, virus-induced gene silencing (VIGS)

## Abstract

Histone acetylation is a dynamic modification process co-regulated by histone acetyltransferases (HATs) and histone deacetylases (HDACs). Although HDACs play vital roles in abiotic or biotic stress responses, their members in *Triticum* *aestivum* and their response to plant viruses remain unknown. Here, we identified and characterized 49 *T. aestivum* *HDACs (TaHDACs)* at the whole-genome level. Based on phylogenetic analyses, *TaHDACs* could be divided into 5 clades, and their protein spatial structure was integral and conserved. Chromosomal location and synteny analyses showed that *TaHDACs* were widely distributed on wheat chromosomes, and gene duplication has accelerated the *TaHDAC* gene family evolution. The cis-acting element analysis indicated that *TaHDACs* were involved in hormone response, light response, abiotic stress, growth, and development. Heatmaps analysis of RNA-sequencing data showed that *TaHDAC* genes were involved in biotic or abiotic stress response. Selected *TaHDACs* were differentially expressed in diverse tissues or under varying temperature conditions. All selected *TaHDACs* were significantly upregulated following infection with the *barley stripe mosaic virus* (BSMV), *Chinese wheat mosaic virus* (CWMV), and *wheat yellow mosaic virus* (WYMV), suggesting their involvement in response to viral infections. Furthermore, *TaSRT1*-silenced contributed to increasing wheat resistance against CWMV infection. In summary, these findings could help deepen the understanding of the structure and characteristics of the *HDAC* gene family in wheat and lay the foundation for exploring the function of *TaHDACs* in plants resistant to viral infections.

## 1. Introduction

Histone N-terminal tails harbor a variety of posttranslational modification sites for acetylation, ubiquitination, sumoylation, methylation, phosphorylation, glycosylation, biotinylation, carbonylation, and ADP-ribosylation [1,2,3]. Histone acetylation is one of the most intensively studied posttranslational modifications, and current research has focused on the mechanisms and functions of histone acetylation [4,5]. It is well known that histone acetylation plays a crucial role in the epigenetic regulation of gene expression in eukaryotic cells. In addition, histone acetylation and deacetylation are dynamic and reversible biological processes that affect chromatin function and structure [6,7]. In response to developmental signals and environmental stimuli, the acetylation of histone lysine residues, which is regulated via the opposing activities of histone acetyltransferases (HATs) and histone deacetylases (HDACs), is quickly triggered [8,9].

In particular, HDACs are key enzymes involved in the acetylation process and are widely distributed in eukaryotes, including yeasts, animals, and plants. The first histone deacetylation gene, now called human *HDAC1*, was isolated and cloned from human Jurkat T cells in 1996 [10]. Since then, at least 18 HDACs involved in gene silencing, transcription, cell cycle, differentiation, DNA replication, and damage repair have been identified in humans. Based on the homology of HDACs in yeast, these HDAC proteins have been divided into three categories: RPD3, HDA1, and SIR2 [11]. In the past 20 years, plant HDACs have received extensive research attention, and an increasing number of HDACs from *Arabidopsis*, rice, maize, barley, and other plants have been identified and characterized [12,13,14]. A total of 18 HDACs have been identified in *Arabidopsis*, 12 of which belong to the RPD3/HDA1-like family, including six members of Class I (HDA6, HDA7, HDA9, HDA10, HDA17, and HDA19), five members of Class II (HDA5, HDA8, HDA14, HDA15, and HDA18), and one member of Class IV (HDA2); two of which belong to SIR2-like family (Class III, SRT1, and SRT2); and four of which belong to plant-specific HD2-type HDACs (HD2A, HD2B, HD2C, and HD2D) [12,15]. Wheat (*Triticum aestivum* L.) is one of the most important crops worldwide [16], as it has robust adaptability and high yield potential and is essential for the survival of humans and animals [17]. However, the structure, function*,* and expression of the members of the *HDAC* gene family in wheat remain unknown.

HDACs are reportedly involved in the abiotic stress response [4,18]. For instance, under high-temperature stress, the roots and shoots of *Arabidopsis hda19-1* mutant plants exhibited a disorderly growth [19]. The expression of *HDACs* in maize was highly induced under cold stress, causing the complete deacetylation of the H3 and H4 histones [20]. Maize treated with the trichostatin A (TSA) HDAC inhibitor under chilling stress strongly inhibited the expression of the *ZmDREB1* and *ZmCOR413* maize cold-responsive genes [20]. After cold acclimation, the freezing tolerance of *Arabidopsis hda6* mutants was found to be significantly lower than that of wild-type plants [21]. In addition, the majority of *HDAC* genes were differentially expressed after salt and drought treatment in rice [22]. These findings suggested that *HDACs* might play essential roles in *Arabidopsis*, maize, and rice in response to abiotic stress, especially low-temperature stress. Therefore, it is particularly important to analyze the relationship between *HDACs* and the temperature response in wheat.

Besides, HDACs are also involved in response to biotic stress in plants. For instance, the transcription of *AtHDA19* was induced in *Arabidopsis* by pathogen-related hormones (jasmonate, JA*,* and ethylene) and the fungal pathogen *Alternaria brassicicola*. Overexpression of *AtHDA19* increased the expression of the ethylene response factor 1 (*ERF1*), a key factor in defense response, and enhanced the resistance of transgenic plants against *A. brassicicola* [23]. Besides, the interaction between WRKY38, WRKY62, and *At*HDA19 resulted in enhanced plant resistance against *Pseudomonas syringae* [24]. The *AtHDA19* mutant was characterized by salicylic acid (SA) accumulation and *PR1* and *PR2* upregulation, which enhanced the plant tolerance to *P. syringae*. In contrast, the *AtSRT2* gene reportedly negatively regulates plant resistance against *P. syringae* by inhibiting SA biosynthesis [25]. In general, HDACs are required in plant defense response to pathogens, and different HDAC family members might play different roles [26]. Although HDACs play diverse roles in plant defense response to invading fungal and bacterial pathogens, they have rarely been implicated with the responsiveness of the plants to viral infection. Therefore, analyzing the expressional changes of *HDAC* family-related genes after the viral infection of wheat would have a certain guiding significance for pathogen-free production of wheat.

In this study, we identified 49 *HDAC* genes in the wheat genome, and analyzed their characteristics, evolutionary relationships, chromosomal location and synteny relationship, protein structure, cis-acting elements, tissue-specific expression levels, response to abiotic or biotic stresses, and expression patterns under temperature variations and viral infection. Silencing *TaSRT1* could improve wheat resistance against *Chinese wheat mosaic virus* (CWMV). This study provided valuable information for the functional investigation of the gene family of *T. aestivum HDACs (TaHDAC)* and helped us screen candidate genes involved in plant resistance against viral infections.

## 2. Materials and Methods

### 2.1. Identification of TaHDAC (Histone Deacetylase) Genes in Triticum aestivum

To recognize *TaHDAC* genes in wheat, according to the *AtHDAC* gene IDs reported in a previous review [15], the amino acid sequences of all *AtHDACs* in *Arabidopsis thaliana* were downloaded from the *Arabidopsis* Information Resource (https://www.arabidopsis.org). Subsequently, these sequences were used as queries to perform BLASTp and tBLASTn searches (E-value < 1.0 × 10^−5^, Identity > 50%) against the wheat reference sequence in the Ensembl Plants database supported by the International Wheat Genome Sequencing Consortium (IWGSC, http://www.wheatgenome.org/). Next, the coding sequence (CDS) length, the number of exons, and chromosomal locations were determined using Ensembl Plants (http://plants.ensembl.org/tools.html). The molecular weight and isoelectric point (pI) of proteins were predicted using ExPASy (https://web.expasy.org/protparam/), and their subcellular location was predicted using the Bologna Unified Subcellular Component Annotator (BUSCA) webserver (http://busca.biocomp.unibo.it/) [27].

### 2.2. Multiple Sequence Alignment and Phylogenetic Analysis

After sequence screening, multiple sequence alignment of the amino acid sequences of *AtHDACs* and *TaHDACs* or *TaHDACs* alone were performed using DNAMAN 6.0 or ClustalW in MEGA7.0 [28,29] with default parameters. The phylogenetic tree was constructed based on the neighbor-joining (NJ) method, and 1000 bootstrap replicates. The data processing used pairwise deletion, while the tree-building model adopted a Poisson distribution.

### 2.3. Chromosomal Locations and Synteny Analysis

To analyze the distribution of *TaHDAC* genes in wheat chromosomes and gene duplication events, the reference information of the wheat genome was downloaded from NCBI (https://www.ncbi.nlm.nih.gov/genome/?term=wheat) and Ensembl Plants database (http://plants.ensembl.org/Triticum_aestivum/Info/Index). Subsequently, the chromosomal location and synteny relationship was identified using TBtools [30].

### 2.4. Protein Structure Prediction

To predict the spatial protein structure, the homology modeling of TaHDAC proteins was performed using the automated SWISS-MODEL homology modeling server (https://swissmodel.expasy.org/) [31].

### 2.5. Presumptive Promoter Cis-Acting Elements

In the putative promoter regions, the 2000 bp sequences upstream of each *TaHDAC* gene were used to identify their cis-acting elements using the PlantCARE online tool (http://bioinformatics.psb.ugent.be/webtools/plantcare/html/). The clustering and arranging of all cis-acting elements were realized with the TBtools [30].

### 2.6. Analysis of the Expression Patterns of TaHDAC Genes by RNA-Seq Datasets

The expression profile datasets of wheat variety “Chinese Spring” were obtained by the Wheat Expression Browser database (http://www.wheat-expression.com/) [32,33]. According to the gene ID, we searched the TaHDAC genes on the website. The expression of TaHDAC genes under different abiotic and biotic stress conditions (including heat, PEG6000, Fusarium graminearum, powdery mildew E09, stripe rust CYR31) was analyzed. Results were visualized as heatmaps using TBtools [30].

### 2.7. Plant Growth and Treatments

Wheat *(Triticum aestivum* L. cv. Yangmai 158) seeds were soaked in distilled water in a glasshouse at 23 °C with a 16 h light/8 h dark photoperiod. After 1 week(wk), wheat seedlings were transplanted into the soil in small black square pots and used for the viral inoculation assay. When wheat seedlings reached the 3-leaf stage, we applied abiotic stress treatment. Then, we selected wheat seedlings of similar size and placed them in climate chambers at 8 °C, 15 °C, 20 °C, or 25 °C, and tested the expression levels of related genes at 10 days.

### 2.8. Foxtail Mosaic Virus (FoMV)-Based Virus-Induced Gene Silencing (VIGS) in Wheat

Foxtail mosaic virus (FoMV)-mediated virus-induced gene silencing (VIGS) had been successfully used in barley and wheat with Nicotiana benthamiana as intermediate host [34]. The specific fragment (300 bp) of TaSRT1 (TraesCS2D02G075800.1) was amplified from the wheat cDNA and then digested with MluI for construction of TaSRT1 inverted-repeats according to the methods in a previous study [34]. The product was cloned into the AscI sites of pFoMV-sg to generate recombinant vector FoMV:TaSRT1, which was then transformed into Agrobacterium tumefaciens strain GV3101. A. tumefaciens containing FoMV:TaSRT1 was cultivated in yeast extract tryptone (YEP) medium with rifampicin (50 μg/mL) and kanamycin (100 μg/mL) at 28 °C for 16 h. After being resuspended in infiltration buffer (10 mM MES, pH 5.6, 10 mM MgCl_2_, 200 mM acetosyringone), the A. tumefaciens was infiltrated into the leaves of N. benthamiana. After 7 days post-inoculation (dpi), the infiltrated leaves were ground in phosphate buffer saline (PBS) for rub-inoculating the 2-leaf stages wheat leaves. The successfully silenced plants were used to inoculate with the CWMV.

### 2.9. Viral Inoculation

The linearized *barley stripe mosaic virus* (BSMV) RNAα, β and γ plasmid transcripts were transcribed in vitro and then mixed in equal amounts at a molar concentration ratio of 1:1:1, with excess inoculation buffer (named FES) (0.06 M potassium phosphate, 0.1 M glycine, 1% bentonite, 1% sodium pyrophosphate decahydrate, 1% celite, pH 8.5) as inoculation buffer, as previously described [35]. Subsequently, the mixtures were inoculated into 2-wk-old wheat seedlings, whereas plants inoculated with FES buffer were used as the negative control.

The linearized plasmids of CWMV or *wheat yellow mosaic virus* (WYMV) RNA1 and RNA2 were transcribed in vitro as previously described [36]. Subsequently, the CWMV or WYMV transcripts were separately mixed into the inoculation buffer, and the 2 mixtures were rub-inoculated to 2-week-old wheat plants using the same method.

### 2.10. RNA Extraction and Real-Time Quantitative Polymerase Chain Reaction (RT-qPCR)

Total RNA was extracted from each wheat sample using a Hipure Plant RNA Mini Kit (Magen, Guangzhou, China), according to the manufacturer’s instructions, and stored at −80 °C until use. The first-strand cDNA was synthesized from 1 μg total RNA per 20 μL reaction volume using a First Strand cDNA Synthesis Kit (Toyobo, Kita-ku, Osaka, Japan), according to the manufacturer’s instructions, and as previously described [37]. The real-time quantitative polymerase chain reaction (RT-qPCR) assay was conducted on an ABI7900HT Sequence Detection System (Applied Biosystems QuantStudio 5, Foster City, CA, USA) using the Hieff qPCR SYBR Green Master Mix (Yeasen, Shanghai, China). At least 3 biological replicates, with 3 technical replicates, were used for each treatment. The relative gene expression levels were calculated according to the 2^−ΔΔC(t)^ method [38]. In each reaction, the *Triticum aestivum* cell division cycle (CDC) gene was used as an internal reference gene [35]. All primers used in RT-qPCR are listed in Table S1.

## 3. Results

### 3.1. Genome-Wide Identification and Characterization of TaHDAC Genes

**Figure 1:**
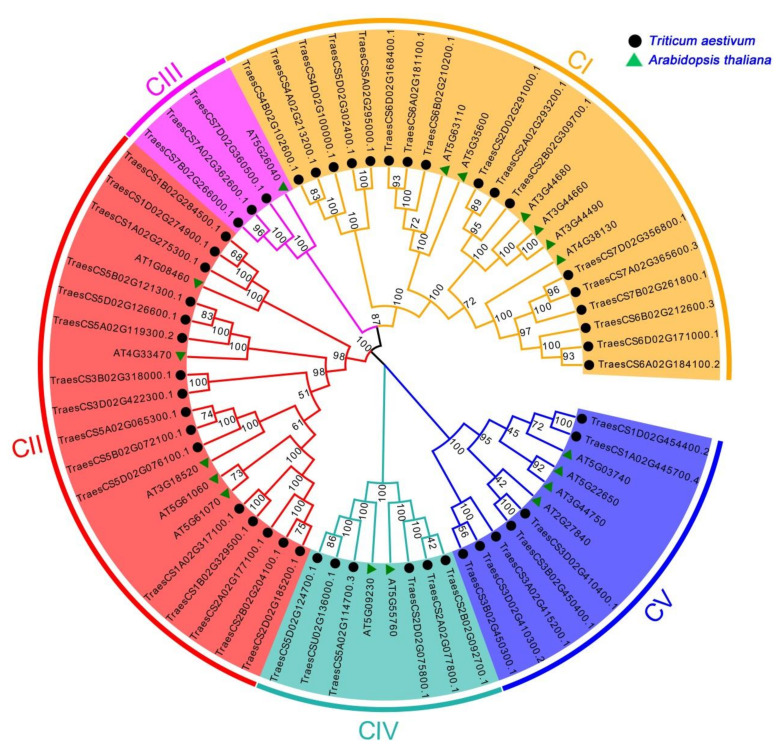
Phylogenetic relationships among histone deacetylase (HDAC) proteins in *Triticum aestivum* and *Arabidopsis thaliana*. All HDAC proteins were allocated into 5 clades. Orange, red, purple, cyan, and blue represent C I, C II, C III, C IV, and C V, respectively. The unrooted tree was established by the neighbor-joining (NJ) method using the MEGA7.0 software with 1000 bootstrap replicates.

To conduct a genome-wide gene identification of the wheat HDAC gene family, we used the *AtHDAC* gene as a query sequence to search for the *TaHDAC* genes in the wheat genome database. Following the genome-wide search of HDACs, we identified a total of 49 full-length *HDAC* homologs in wheat. Detailed information about *TaHDACs*, such as gene ID, location, physical and chemical properties, were listed in Table 1. The lengths of the CDS regions were distributed from 930 to 2082 bp, with the encoded sequences ranging from 309 to 693 aa. The relative molecular weight of the proteins ranged from 33.16 to 74.54 kDa, the pI varied from 4.60 to 9.42, while the number of exons varied from 1 to 17. The prediction results of the subcellular localization indicated that *TaHDAC* genes were located in the nucleus, cytoplasm, chloroplast, mitochondria, and extracellular space. Previous research has shown that the *AtHDAC* gene family could be divided into 3 categories in *Arabidopsis,* including the RPD3/HDA1-like, SIR2-like, and HD2 families [15,26]. Based on multiple sequence alignment results of *AtHDACs* and *TaHDACs*, we created an evolutionary tree and divided them into 5 clades (named C I, C II, C III, C IV, and C V) (Figure 1). Among them, C I, C II, and C III were identified to belong to the RPD3/HDA1-like family, whereas C IV and C V represented the SIR2-like and HD2 families, respectively. We observed that the phylogenetic distribution of the members of *TaHDACs* in different clades was not uniform; C I included 6 members from *Arabidopsis thaliana* and 17 members from *Triticum aestivum*, C II contained 5 members from *Arabidopsis thaliana* and 16 members from *Triticum aestivum*, C III consisted of 1 member from *Arabidopsis thaliana* and 3 members from *Triticum aestivum*, C IV included 2 members from *Arabidopsis thaliana* and 6 members from *Triticum aestivum*, and C V comprised 4 members from *Arabidopsis thaliana* and 7 members from *Triticum aestivum*. In addition, we created an individual phylogenetic tree of the *TaHDACs* to examine their respective phylogenetic relationships (Figure S1).

### 3.2. Chromosomal Location and Synteny Analysis of TaHDAC Genes in Wheat Chromosomes

**Figure 2:**
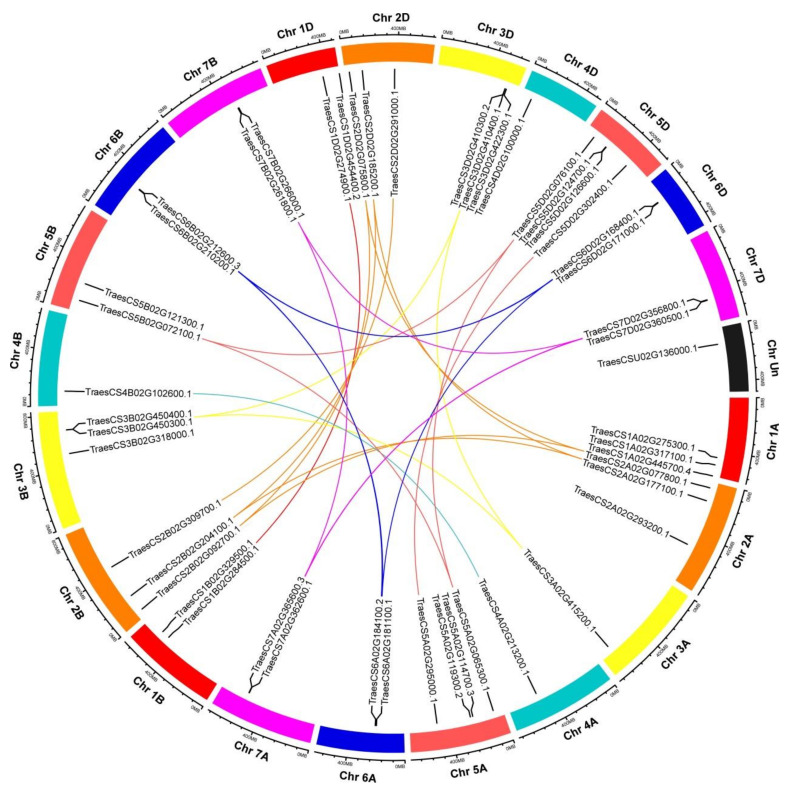
Distribution and duplication of *TaHDAC* genes in wheat chromosomes. Chromosome columns of the same chromosome group are represented by the same color, with different colors distinguishing different chromosome groups. The bold-colored curve indicates each gene pair subjected to gene duplication, and the short black lines indicate the location of these genes on chromosomes. The graphs of chromosomal location and synteny analysis were established using TBtools.

Based on the initiation position of each gene on wheat chromosomes, 48 *TaHDAC* genes were unevenly distributed on the 21 chromosomes (chromosome 1A to 7D, Chr1A to Chr7D). Interestingly, *TraesCSU02G136000.1* was identified in unknown wheat chromosome (ChrUn). Chr5 contains the largest number of *TaHDAC* genes (10), whereas Chr4 contains the fewest number of *TaHDAC* genes (3). 9 *TaHDAC* genes were detected on Chr2. 7 *TaHDAC* genes were found on Chr1 and Chr3, respectively. Six *TaHDAC* genes were found on Chr6 and Chr7, respectively (Figure 2).

In order to determine whether *TaHDAC* genes have a duplication relationship during the process of evolution, we conducted a synteny analysis based on the location of each gene in wheat chromosomes. Following this analysis, 21 *TaHDAC* genes (*TraesCS3D02G410300.2/410400.1/422300.1*,) *TraesCS3B02G450300.1*/*450400.1*, *TraesCS5A02G114700.3/119300.2*, *TraesCS5D02G124700.1/126600.1*, *TraesCS6A02G181100.1/184100.2*, *TraesCS6B02G210200.1/212600.3*, *TraesCS6D02G168400.1/171000.1*, *TraesCS7A02G362600.1/365600.3*, *TraesCS7B02G261800.1/266000.1*, *TraesCS7D02G356800.1/360500.1*) were clustered into ten tandem duplication event regions on Chr3B/3D/5A/5D/6A/6B/6D/7A/7B/7D (Figure 2 and Table S2). The paralogous *TaHDACs* on different chromosomes were segmental duplication events and clustered together.

### 3.3. Protein Structure Prediction of 10 TaHDACs

**Figure 3:**
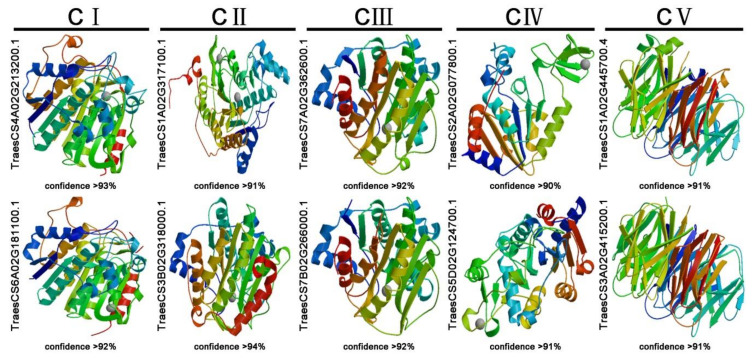
Prediction of the spatial structure of TaHDACs. The 10 TaHDACs with high confidence (greater than 90%) were displayed.

It is widely accepted that there is a close correlation between the spatial structure and function of a protein, as the function of a protein is achieved through changes in its spatial conformation. To further determine the spatial structure of TaHDACs, we used the SWISS-MODEL website to conduct homology modeling. All 49 TaHDACs could be forecasted as models, indicating that they maintained their structural integrity, which plays an important role in their function, during the evolutionary process. In each clade, we selected 2 proteins with the highest confidence level and coverage (greater than 90%) (TraesCS4A02G213200.1 and TraesCS6A02G181100.1 in C I, TraesCS1A02G317100.1, and TraesCS3B02G318000.1 in C II, TraesCS7A02G362600.1 and TraesCS7B02G266000.1 in C III, TraesCS2A02G077800.1, and TraesCS5D02G124700.1 in C IV, TraesCS1A02G445700.4, and TraesCS3A02G415200.1 in C V). Our results are illustrated in Figure 3. At the same time, we found that the spatial conformation of proteins belonging to the same clade exhibited a high degree of similarity.

### 3.4. Prediction of Cis-Acting Elements in the 49 TaHDACs

**Figure 4:**
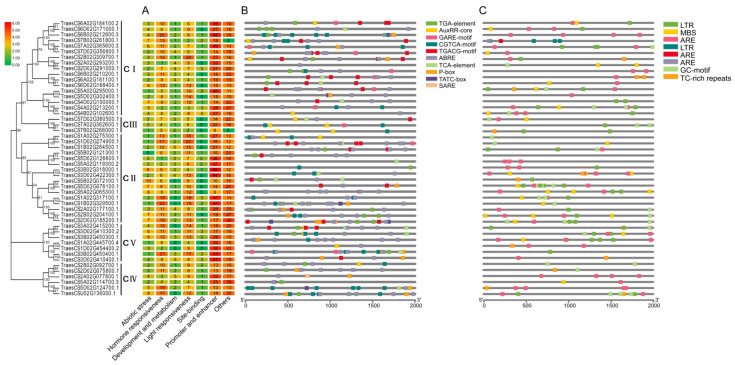
Predicted cis-acting elements in *TaHDAC* genes. (**A**) All cis-acting elements are divided into 7 classifications; the number of cis-acting elements in the promoter region of each *TaHDAC* gene is counted. (**B**) The cis-acting elements related to auxin (IAA), gibberellin (GA), methyl jasmonate (MeJA), abscisic acid (ABA), and salicylic acid (SA) identified in the promoter region of each *TaHDAC* gene. (**C**) The abiotic stress-related cis-acting elements identified in the promoter region of each *TaHDAC* gene.

The cis-acting elements predicted in the promoter regions of the 49 *TaHDAC* genes could be divided into 7 categories (Figure 4). Following analysis, we predicted a total of 58 cis-acting elements in the promoter regions of *TaHDACs*. Among all recognized elements, the most abundant was shown to be the light-responsive type, with 27 hits. Additionally, we identified 5 abiotic stress-response elements, 10 hormone response elements, 9 development and metabolism response elements, 4 site-binding elements, 2 promoter and enhancer elements, and 1 other element. Studies have reported that *HDAC* genes play a role in the plant response to abscisic acid (ABA) and SA [25,39]. To investigate whether *TaHDACs* have an analogous function, we used PlantCARE to conduct promoter analysis and identified some cis-acting elements that respond to auxin (IAA), gibberellin (GA), methyl jasmonate (MeJA), ABA, and SA. We noted that the IAA response element mainly included the TGA-element and AuxRR-core, the GA responsive element contained a TATC-box, P-box, and GARE-motif, the MeJA response element covered a CGTCA-motif and TGACG-motif, the ABA response element consisted of ABRE, and the SA response element incorporated a TCA-element and SARE. Among the predicted abiotic stress response elements, the anaerobic induction-related components identified were ARE and GC-motif, LTR, and MBS elements participating in low-temperature response and drought induction, respectively. In addition, we also predicted TC-rich repeat elements involved in defense and stress responsiveness. Finally, an evolutionary analysis revealed that these response elements were unevenly distributed in each clade.

### 3.5. Tissue-Specific Expression of TaHDACs

**Figure 5:**
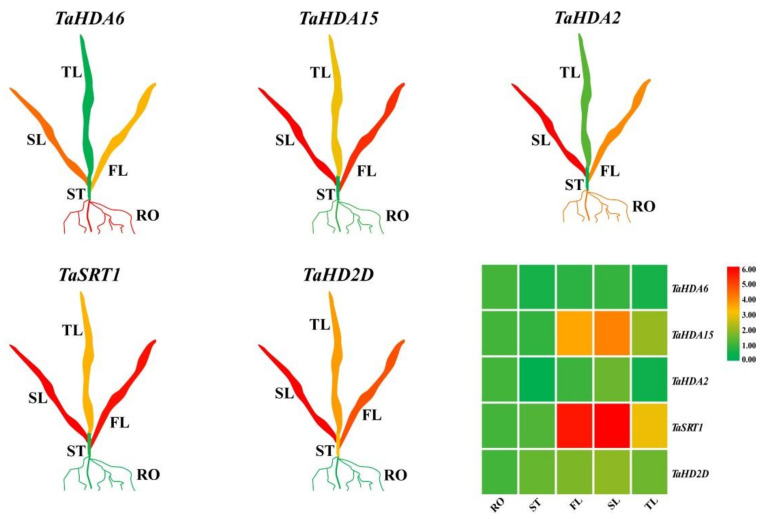
The expression level of representative *TaHDAC* genes in different tissues using real-time quantitative polymerase chain reaction (RT-qPCR) analysis. RO: root; ST: stem; FL: first leaf; SL: second leaf; TL: third leaf. The relative expression level of each biological sample was calculated based on 3 biological and 3 technical replicates. Red represents a high expression value, whereas green represents a low expression value. The relative expression level of *TaHDAC* genes was visualized using TBtools.

To explore the biological functions of *TaHDACs* in wheat, we randomly selected a gene from each clade (C I–C V) to analyze their respective expression level in the root, stem, first, second, and third leaves using RT-qPCR analysis (Figure 5 and Figure S2). All selected genes had different expression levels in each tissue. Taking the root as a control, the expression level of *TaHDA6* (*TraesCS6A02G181100.1*) in C I was found to be downregulated in each tissue. In contrast, the expression level of *TaHDA15* (*TraesCS5D02G076100.1*) in C II was higher, except for its expression level in the stem. The expression levels of *TaSRT1* (*TraesCS2D02G075800.1*) and *TaHD2D* (*TraesCS3B02G450300.1*) in C IV and V were shown to be upregulated in various tissues. The expression level of *TaHDA2* (*TraesCS7B02G266000.1*) in C III was observed to be upregulated in the first leaf, whereas its expression level in the second leaf showed no significant change and was downregulated in the stem and the third leaf.

### 3.6. Expression Patterns of TaHDAC Genes in Response to Abiotic and Biotic Stresses

**Figure 6:**
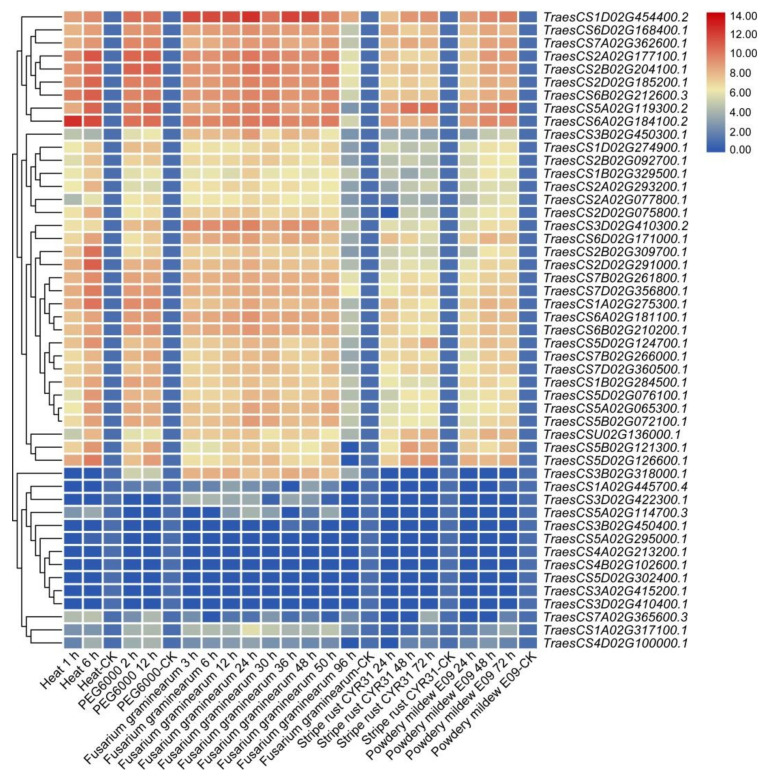
The expression profiles of the *TaHDAC* gene family in *Triticum aestivum* under abiotic or biotic stresses. The heatmaps were constructed by TBtools based on the expression datasets. Expression levels were represented in diverse colors, with red indicating higher expression levels and blue indicating lower expression levels.

To further clarify the underlying functions of TaHDAC genes response to stresses, the expression profiles were analyzed under heat, PEG6000, Fusarium graminearum, powdery mildew E09, or stripe rust CYR31 treatments using RNA-seq database (Wheat Expression Browser database). After calculation and analysis based on the FPKM values of TaHDAC genes under different stresses and drew the heatmaps (Figure 6). Our results showed that most TaHDAC genes were involved in abiotic and biotic stress responses. The numbers in same group, such as TraesCS2A02G177100.1, TraesCS2B02G204100.1, TraesCS2D02G185200.1, and TraesCS6B02G212600.3, showed similar expression patterns under abiotic or biotic stresses. In addition, the expression of TraesCS6A02G184100.2 was strongly induced under heat treatment at 1 or 6 h post-treatment (hpt). TraesCS2A02G177100.1 and TraesCS2B02G204100.1 were upregulated under PEG6000 at 2 or 12 hpt. TraesCS1D02G454400.2 showed a higher expression level under Fusarium graminearum treatment, indicating it might be involved in response to pathogens. Interestingly, almost all TaHDAC genes showed high expression levels under powdery mildew E09 treatment, whereas others were reduced. These results suggested that TaHDAC genes participate in a variety of abiotic or biotic stress responses in T. aestivum.

### 3.7. Expression Patterns of TaHDACs under Temperature Gradients

**Figure 7:**
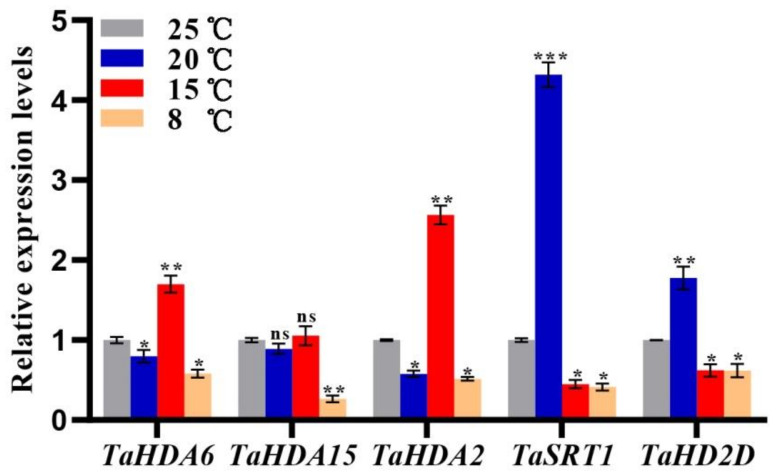
RT-qPCR results of the expression levels of selected *TaHDAC* genes under diverse temperature conditions. The mRNA expression level of each gene in wheat seedlings is presented as the mean ± standard deviation (SD) from 3 biological samples, with each biological sample having 4 technical duplicates. Statistical analyses were performed using the Student’s *t*-test. Asterisks indicate a significant difference when compared with the control. *, *p* < 0.05; **, *p* < 0.01; ***, *p* < 0.001; ns, no significant difference.

Based on cis-acting elements and expression profiles analysis, we found that the *TaHDAC* genes might be involved in temperature response. To better understand the mRNA expression level of *TaHDACs* in wheat under different temperature conditions, we isolated the total RNA from wheat plants grown in a climate chamber (8, 15, 20, or 25 °C) at 10 d, and determined the relative expression of representative *TaHDACs* using RT-qPCR analysis (Figure 7). We found that all 5 selected genes were up- or down-regulated under different temperatures, although the expression of *TaHDA15* under 20 or 15 °C was not significant change compared to the control (25 °C). However, we could observe several exceptions, such as the expression of *TaHDA6/2* at 15 °C were significantly upregulated, whereas them expression level were decreased at 8 °C. Interestingly, the expression of *TaSRT1* was shown to be the highest at 20 °C. Likewise, under this temperature condition, the expression of *TaHD2D* was also increased as expected.

### 3.8. Expression Patterns of TaHDACs under Viral Infection

**Figure 8:**
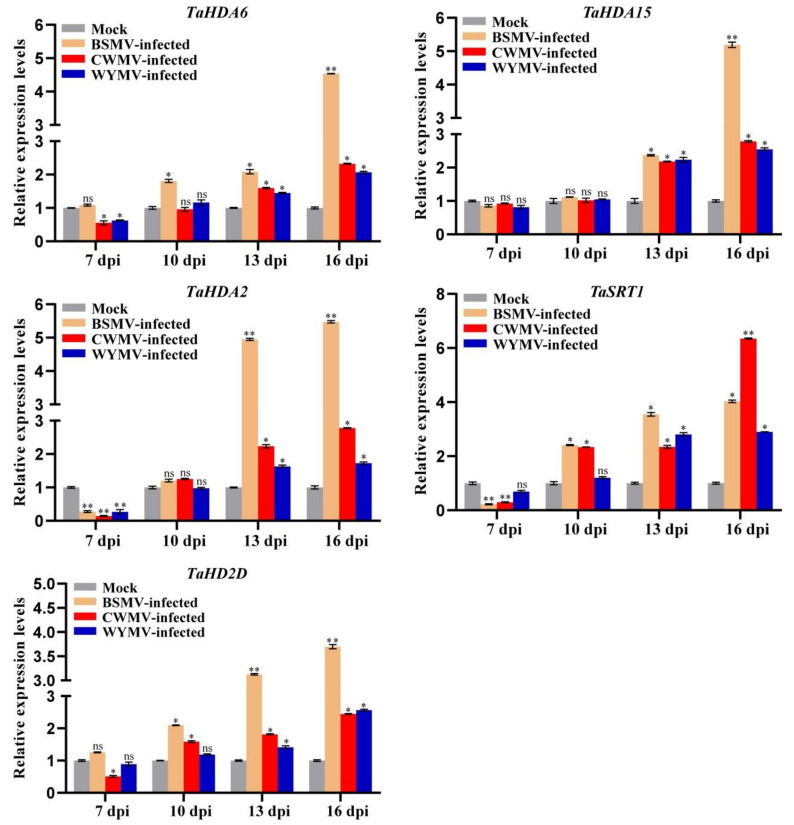
RT-qPCR results of the expression level of representative *TaHDAC* genes following inoculation of wheat plants with different viruses. The relative expression levels were calculated from 3 independent biological replicates using the 2^−ΔΔC(t)^ method. *TaCDC* was used as internal control. Statistical analyses were performed using the Student’s *t*-test. *, *p* < 0.05; **, *p* < 0.01; ns, no significant difference.

Although RNA-seq datasets have shown that *TaHDAC* genes participate in the stress response to pathogens, it is unknown whether *TaHDACs* respond to viral infection. To investigate whether *TaHDACs* respond to plant viral infection, the second leaf (bottom-up) was collected and used in RT-qPCR analysis to study the relative expression patterns of *TaHDACs* in wheat plants following infection by BSMV, CWMV, and WYMV (Figure 8). Accordingly, 7 to 16 d post-inoculation (dpi) with the BSMV virus, the expression of almost all *TaHDACs* tested was shown to be increased relative to the mock (FES buffer), especially that of *TaHDA6/2/2D*. Strikingly, *TaHDA2* expression was significantly increased after BSMV-infection at 13 and 16 dpi (greater than 4.9-fold and 5.4-fold, respectively, while that of *TaHDA6/15* was also significantly increased at 16 dpi (greater than 4.5-fold and 5.1-fold, respectively). Furthermore, the expression levels of *TaHDACs* tested after infection of wheat plants with CWMV or WYMV showed an upward trend from 7 to 16 dpi. Subsequently, we also noticed the strongly induced expression (greater than 6.3-fold) of *TaSRT1* after CWMV infection at 16 dpi.

### 3.9. Silencing TaSRT1 Attenuates Chinese Wheat Mosaic Virus (CWMV) Infection in Wheat

**Figure 9:**
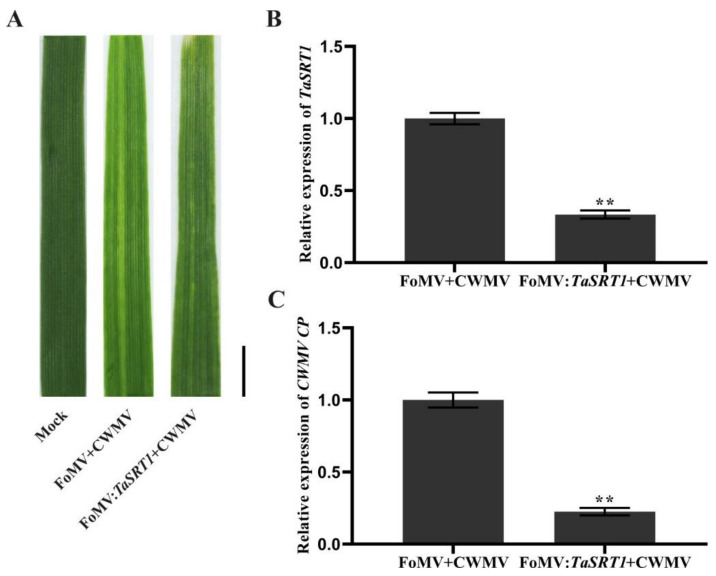
Silencing *TaSRT1* by *Foxtail mosaic virus* (FoMV)-based virus-induced gene silencing (VIGS) significantly alleviated *Chinese wheat mosaic virus* (CWMV) infection in *Triticum aestivum*. (**A**) Phenotypes in newly formed leaves of the plants inoculated with phosphate buffer saline (PBS) as Mock, FoMV + CWMV, FoMV:*TaSRT1* + CWMV, respectively. Photographs were taken at 14 d post-inoculation (dpi) with viruses. Bar = 1 cm. (**B**) Relative expression level of *TaSRT1* was analyzed with FoMV:*TaSRT1* + CWMV infected wheat plants by RT-qPCR. Total RNA from a FoMV + CWMV infected wheat plants was isolated used as a control. (**C**) Relative expression level of *CWMV CP* was analyzed in leaf 4 from *TaSRT1*-silenced or non-silenced wheat plants. Each experiment was performed using three biological and each biological sample had three technical replicates. Statistical analyses were performed using the Student’s *t*-test. **, *p* < 0.01.

After analyzing the changes of these selected genes under viral infection, we found that the expression of TaSRT1 gradually increased following CWMV infection and reached the highest expression level at 16 dpi, suggesting that it might play a significant role in the process of CWMV infection. To investigate the role of TaSRT1 in CWMV resistance, we used FoMV-based VIGS technology to silence TaSRT1 in wheat in order to verify its biological function. We inoculated 6 two-leaf stage wheat seedlings with saps of N. benthamiana leaves agroinfiltrated with FoMV + CWMV or FoMV:TaSRT1 + CWMV, respectively. All wheat plants infected with FoMV + CWMV or FoMV:TaSRT1 + CWMV usually showed mosaic symptoms in newly formed leaves, and compared to the FoMV + CWMV plants, TaSRT1-silenced plants exhibited milder symptoms (Figure 9A). Furthermore, the silencing level of the TaSRT1 gene in the FoMV:TaSRT1 + CWMV co-inoculated wheat seedlings through RT-qPCR using TaSRT1 specific primers (Table S3). The result indicated that the TaSRT1 transcript level in the plants co-inoculated with FoMV:TaSRT1 + CWMV were better silenced (p < 0.01) than the plants co-inoculated with FoMV + CWMV (Figure 9B). Following this, the expression level of CWMV CP was also detected via RT-qPCR using CP specific primers (Table S1) in these plants. The results indicated that the expression level of CWMV CP was detected via RT-qPCR, and the CWMV CP expression level of FoMV:TaSRT1 + CWMV inoculated wheat was significantly reduced compared to the inoculated wheat with FoMV + CWMV (Figure 9C). These results suggest that silencing TaSRT1 contributes to the improvement of host plant resistance to the virus.

## 4. Discussion

The HDAC proteins reportedly play essential roles in regulating chromatin structure, gene expression, plant growth, development, and stress responses [9,26,40]. The *HDAC* gene family has been identified and characterized in most organisms, including archaebacteria, eubacteria, fungi, and animals (such as fruit-fly, mice, chicken, and humans) [11,41,42], as well as in several plants such as pea [43], *Arabidopsis*, maize, rice, barley [14], potato [44], grape [45], and tobacco [46]. However, to date, there have been few studies on HDACs in wheat. Recent advances in sequencing and annotation of the allohexaploid wheat genome [47,48,49] have provided favorable conditions for revealing the evolutionary traits, organization, and expression of the wheat *HDAC* gene family the whole-genome level. This study systematically identified 49 HDACs within the *Triticum aestivum* genome and performed genome-wide identification, phylogenetic analysis, determination of chromosomal locations, and synteny relationships’ protein spatial structures, cis-acting elements, and expression patterns under diverse stress treatments. The *AtHDAC* gene family in *Arabidopsis*, includes 3 subfamilies: the RPD3/HDA1-like, SIR2-like, and HD2 families [15,26], consistent with the *TaHDACs* in wheat. Based on sequence similarity, we further divided the RPD3/HDA1-like family into 3 clades. In general, using phylogenetic analysis in *Arabidopsis*, these *TaHDACs* could be divided into 5 clades (Figure 1). Our results showed that the wheat genome harbors a higher number of *HDAC* genes compared with those identified in *Arabidopsis*, especially in the RPD3/HDA1-like subfamily (consisting of C I, C II, and C III clades) (Table S3); these results imply that there are large sequence variations and biological function differences within the *TaHDAC* gene family. Gene duplication events are critical to the expansion of gene families and genome evolution or rearrangement, mainly due to tandem, segment, and transposition duplication [50,51,52]. As the results showed, we found that 21 *TaHDAC* genes exhibited a synteny relationship, and these paralogous gene pairs were unevenly distributed on each wheat chromosome (Figure 2 and Table S1), which likely contributes to the expansion of the *TaHDAC* gene family. Of note, gene duplication events also contributed to the development of new biological functions during wheat genome evolution. Moreover, the highly similar protein spatial structures also implied that *TaHDACs* are evolutionarily conserved, and clarify the three-dimensional structures of proteins could provide valuable information to analyze the functions of TaHDAC in plants. (Figure 3).

The HDAC proteins are widely expressed in almost all types of plant tissue, such as vegetative tissue, callus, seeds, flowers, and roots. The available microarray analysis data indicated that 16 *Arabidopsis HDAC* genes were detected to be expressed in 79 different tissues [12]. Both HD2 and RPD3-like subfamily *HDACs* were demonstrated to be highly expressive in inflorescences and young floral tissues, but their expression in vegetative tissues was found to be low. The expression profiles of RPD3-like and HD2 subfamilies of *HDAC* genes were shown to be similar. However, the expression patterns of *AtSRT1* and *AtSRT2* markedly differed [26]. In contrast, the *HDAC* genes in rice were shown to be differentially expressed in various tissues. For example, *OsHDA703* was mainly expressed in calli and seeds, whereas *OsHDA710* was specifically expressed in seedlings and stamens. Besides, both *OsHDA706* and *OsHDA714* were highly expressed in shoots and leaves [22]. In maize, *ZmHDA101* was shown to be expressed throughout the germination process, whereas *ZmHDA108* was expressed when the cell cycle entered the S-phase [53]. These studies suggested that *HDACs* might be involved in different cell dynamic processes, playing vital roles in various plant species. In our study, all *TaHDAC* genes tested were found to be differentially expressed in each tissue (Figure 5). The expression level of all 5 genes in the stem was lower than that in the control (root), indicating that these genes might not participate in stem meristem development. Many *TaHDACs* (*TaHDA15*, *TaSRT1*, and *TaHD2D*) were highly expressed in leaf tissues. Moreover, the expression of *TaHDA6* and *TaHDA2* was demonstrated to be suppressed in the second and third leaves, and stem. In summary, these results implied that *TaHDACs* might play disparate roles in the growth and development of wheat seedlings.

It has been previously demonstrated that *HDACs* are involved in the response of plants to abiotic stress [38,54]. The expression of *HDAC* genes in rice was shown to be regulated by stress-related hormones such as SA, JA, or ABA [22,54]. The expression of *HDA6* and *HDA19* in *Arabidopsis* was also found to be induced by JA [23], whereas the expression of *HD2A*, *HD2B*, *HD2C*, and *HD2D* was shown to be inhibited by ABA and NaCl [39,55]. The *Arabidopsis HDA19* has been associated with the ERF3/4/7 transcription repressors in regulating gene expression in response to abiotic stress [56,57]. In the *axe1-5 AtHDA6* mutant and *AtHDA6-RNAi* plants, the expression of JA response genes (*PDF1.2*, *VSP2*, *JIN1*, and *ERF1*) was reported to be repressed, suggesting that *AtHDA6* was a candidate factor for JA response genes [58]. However, it remained unclear whether the *HDAC* gene family in wheat contained plant hormones and temperature response elements. Thus, to better understand the gene expression patterns, we also analyzed the cis-acting elements of *TaHDAC* promoter regions. Our results showed that the *TaHDAC* promoter regions not only contained phytohormones (IAA, GA, MeJA, ABA, and SA) but also response elements to abiotic stresses, especially temperature response elements (Figure 4). Thus, these outcomes indicating that *TaHDAC* genes might have an essential function in plant growth, development, and coping with abiotic stress. In addition, based on RNA-seq data, we found that *TaHDAC* genes were differently expressed under biotic or abiotic stress (Figure 6), which suggested that *TaHDACs* might be involved in a variety of biological processes in wheat. Previous studies have indicated that the transcription level of the *Arabidopsis AtHDA6* was upregulated at low temperature (2 °C), and compared with the wild type, the *hda6* mutant *axe1-5* was susceptible to freezing temperatures (−18 °C) after cold acclimation [21]. It was further reported that the expression of *HDACs* in maize was induced by cold stress, resulting in the overall deacetylation of H3 and H4 histones [20]. In this study, we found that both *TaHDA6/2* were upregulated at 15 °C compared with control (25 °C); in particular, the *TaSRT1* was significantly upregulated under treatment at 20 °C, suggesting that this gene have a strong response under 20 °C treatment. Moreover, the expression of *TaHDA15* at 20 or 15 °C did not exhibit any significant changes, indicating that it was not sensitive to the 2 temperature conditions (Figure 7). In general, we observed that the expression levels of these *TaHDACs* varied under diverse temperature conditions, consistent with the results of predicted temperature response cis-acting elements, and implying that *TaHDACs* might differ widely in the response of wheat plants to temperature variations, with *TaHDAC* genes in different subfamilies playing different roles.

In addition, studies have reported that *HDACs* are involved in the transcriptional regulation of plant defense responses [23,59]. For example, *Arabidopsis* COL1, which is known to be required for JA response, was shown to interact with *At*HDA6 to promote the defense response of plants to insect pests and pathogens [60]. The *Arabidopsis AtHDA19* could be induced by the *Alternaria brassicicola* fungal pathogen [23] and the *Pseudomonas syringae* bacterial pathogen [24]. However, the expression of *TaHDACs* in wheat following viral infection was largely unknown. Our results indicated that almost all *TaHDACs* tested were upregulated following infection with BSMV, CWMV, and WYMV at 7, 10, 13, and 16 dpi, while with the extension of the viral infection time, the expression level of these genes was shown to be gradually increased (Figure 8). Altogether, these findings indicate that these *TaHDACs* might potentially play a role in the post-infection response. In addition, the results of our RT-qPCR analysis were not inconsistent. For instance, the expression of *TaHDA2* was shown to be decreased after infection with the virus at 7 dpi, implying that this gene might not produce a strong stress response in the early stage of the viral infection. Interestingly, we found that the expression of *TaSRT1* under CWMV infection gradually increased and reached the highest level at 16 dpi. Therefore, we predicted that *TaSRT1* might play an important role in the process of CWMV infection in wheat. Subsequently, we used VIGS to transiently silence *TaSRT1* in wheat to study its biological function under CWMV infection. Studies have shown that knocked down *TaHDT701*, *TaHDA6* and *TaHOS15* enhances the infection of wheat by powdery mildew [61,62]. However, other research has proved that knocked out *AtSRT2* enhanced the resistance of *Arabidopsis* against *Pseudomonas syringae* pv. *tomato* DC3000 (*Pst* DC3000) and induced the expression of PR1 [25]. Consistent with this finding, *TaSRT1*-silenced wheat plants inhibited CWMV infection. Therefore, these results further indicated that the HDAC members in different subfamilies of *T. aestivum* might possess reverse functions under pathogen infection.

## 5. Conclusions

In this study, we identified 49 *TaHDACs* at the genome-wide level, which could be divided into 5 clades. After analysis of chromosome localization and synteny relationship, we found that *TaHDACs* were unevenly distributed in the wheat genome, with 21 *TaHDAC* genes being tandem duplication events, which play a primary role in the expansion of the *T. aestivum HDAC* gene family. All *TaHDACs* belonging to the same subfamily had similar protein spatial structures. Results of analysis of cis-acting elements indicated that *TaHDACs* were involved in hormone response, light response, abiotic stress, and growth and development processes. Analysis of *TaHDAC* gene expression profiles showed that they were involved in heat, PEG6000, *Fusarium graminearum*, powdery mildew and stripe rust responses. The expression patterns of the selected *TaHDAC* genes were shown to be differentially expressed in diverse wheat tissues or under different temperature conditions, and all tested *TaHDACs* were found to be upregulated following infection with BSMV, CWMV, and WYMV. In addition, the silencing of *TaSRT1* enhanced the resistance of wheat against CWMV. These results revealed the *HDAC* gene family members in wheat and indicated their potential functions in plant resistance to viral infections.

## Figures and Tables

**Table 1:** Detailed information about 49 predicted HDACs in *Triticum aestivum.*

Gene ID	Location	CDS Length (bp)	Size (aa)	MW (kDa)	pI	Exons	Predicted Location
*TraesCS1A02G275300.1*	1A:469374580-469377056	1176	391	42.23	5.41	4	nucleus
*TraesCS1A02G317100.1*	1A:508627551-508633273	1194	397	43.38	5.87	9	chloroplast
*TraesCS1A02G445700.4*	1A:593397528-593400047	930	309	33.16	4.69	9	nucleus
*TraesCS1B02G284500.1*	1B:493871055-493873999	1182	393	42.47	5.54	4	nucleus
*TraesCS1B02G329500.1*	1B:555624218-555635513	1173	390	42.83	6.50	9	chloroplast
*TraesCS1D02G274900.1*	1D:370350086-370352713	1101	366	39.48	5.29	4	nucleus
*TraesCS1D02G454400.2*	1D:495110924-495114039	936	311	33.61	4.65	6	nucleus
*TraesCS2A02G077800.1*	2A:35492638-35498995	1323	440	48.72	9.02	13	nucleus
*TraesCS2A02G177100.1*	2A:136335596-136344608	2082	693	74.30	5.15	13	extracellular space
*TraesCS2A02G293200.1*	2A:504284771-504290294	1293	430	49.18	4.98	14	cytoplasm
*TraesCS2B02G092700.1*	2B:53464614-53473185	1398	465	51.53	8.93	15	nucleus
*TraesCS2B02G204100.1*	2B:183653818-183662786	2082	693	74.10	5.18	13	extracellular space
*TraesCS2B02G309700.1*	2B:442785651-442791643	1293	430	49.22	4.98	14	cytoplasm
*TraesCS2D02G075800.1*	2D:32472578-32481962	2037	678	74.54	8.79	16	nucleus
*TraesCS2D02G185200.1*	2D:130425457-130434385	2082	693	73.86	5.22	13	extracellular space
*TraesCS2D02G291000.1*	2D:373215743-373221603	1293	430	49.16	4.98	14	cytoplasm
*TraesCS3A02G415200.1*	3A:658650984-658654457	1212	403	43.60	8.78	10	nucleus
*TraesCS3B02G318000.1*	3B:512513775-512515566	1143	380	41.42	5.81	7	cytoplasm
*TraesCS3B02G450300.1*	3B:690854722-690858308	1152	383	41.59	8.60	10	nucleus
*TraesCS3B02G450400.1*	3B:690859082-690862087	1137	378	40.33	4.63	10	nucleus
*TraesCS3D02G410300.2*	3D:523703723-523707098	1095	364	39.41	8.81	11	nucleus
*TraesCS3D02G410400.1*	3D:523708265-523711340	1299	432	46.11	4.60	9	nucleus
*TraesCS3D02G422300.1*	3D:534235224-534237524	984	327	35.84	5.11	8	cytoplasm
*TraesCS4A02G213200.1*	4A:511187957-511189477	1416	471	51.41	5.32	2	nucleus
*TraesCS4B02G102600.1*	4B:108669975-108671813	1416	471	51.45	5.37	2	nucleus
*TraesCS4D02G100000.1*	4D:77120156-77121980	1416	471	51.56	5.58	2	nucleus
*TraesCS5A02G065300.1*	5A:70489613-70498580	1845	614	66.09	6.00	17	nucleus
*TraesCS5A02G114700.3*	5A:229763694-229772647	1245	414	46.17	9.20	7	nucleus
*TraesCS5A02G119300.2*	5A:248528260-248532633	1335	444	47.94	6.31	9	chloroplast
*TraesCS5A02G295000.1*	5A:503667634-503669320	1455	484	54.55	5.90	1	nucleus
*TraesCS5B02G072100.1*	5B:83785074-83793761	1845	614	66.14	5.71	17	nucleus
*TraesCS5B02G121300.1*	5B:216399368-216404020	1362	453	48.98	5.96	9	extracellular space
*TraesCS5D02G076100.1*	5D:75357410-75367639	1839	612	65.98	5.72	17	nucleus
*TraesCS5D02G124700.1*	5D:190263056-190268180	1191	396	43.78	9.42	12	mitochondrion
*TraesCS5D02G126600.1*	5D:193511197-193521308	1335	444	47.81	6.27	9	chloroplast
*TraesCS5D02G302400.1*	5D:398576928-398578415	1488	495	55.76	6.34	1	nucleus
*TraesCS6A02G181100.1*	6A:206004364-206008422	1377	458	50.97	5.26	6	cytoplasm
*TraesCS6A02G184100.2*	6A:214563801-214569359	1560	519	58.05	5.13	7	nucleus
*TraesCS6B02G210200.1*	6B:277612837-277616723	1377	458	50.99	5.36	6	cytoplasm
*TraesCS6B02G212600.3*	6B:281226434-281231988	1563	520	58.23	5.12	7	nucleus
*TraesCS6D02G168400.1*	6D:153807374-153811200	1377	458	50.97	5.26	6	cytoplasm
*TraesCS6D02G171000.1*	6D:157583544-157589113	1725	574	64.25	5.54	7	extracellular space
*TraesCS7A02G362600.1*	7A:536980277-536984430	1068	355	39.18	6.15	13	chloroplast
*TraesCS7A02G365600.3*	7A:539543850-539554190	1572	523	58.40	5.47	7	nucleus
*TraesCS7B02G261800.1*	7B:482237779-482248552	1560	519	58.00	5.43	7	nucleus
*TraesCS7B02G266000.1*	7B:488410536-488414496	1062	353	39.01	6.09	14	chloroplast
*TraesCS7D02G356800.1*	7D:459923113-459936250	1560	519	58.04	5.33	7	nucleus
*TraesCS7D02G360500.1*	7D:463261509-463265496	1062	353	38.99	6.26	13	chloroplast
*TraesCSU02G136000.1*	Un:121734084-121741419	1191	396	43.75	9.21	11	mitochondrion

## Data Availability

The expression profile datas of *TaHDAC* genes under different stresses is downloaded by the Wheat Expression Browser database (http://www.wheat-expression.com/).

## Supplementary Materials

The following are available online at https://www.mdpi.com/2223-7747/10/1/19/s1, Table S1: List of primers used for this study; Table S2: *TaHDACs* gene duplication pairs (synteny relationship) in wheat *HDAC* gene family; Table S3: The distribution of *HDACs* in *Arabidopsis thaliana* and wheat; Table S4: Amino acid sequences of *Arabidopsis thaliana* and *Triticum aestivum HDACs* used in this study; Table S5: Cis-acting elements of *Triticum aestivum HDACs* in promoter regions; Figure S1: An individual phylogenetic tree was constructed with 49 *Ta*HDACs. All *Ta*HDACs were divided into 5 clades. Orange, red, purple, cyan, and blue represented C I, C II, C III, C IV, and C V, respectively. The unrooted tree was established by the NJ method using MEGA7.0 software with 1000 bootstrap replicates; Figure S2: The expression levels of representative *TaHDACs* in diverse tissues by RT-qPCR (raw data). RO: root; ST: stem; FL: first leaf; SL: second leaf; TL: third leaf. The relative expression level of each biological sample was calculated with three biological replicates and three technical replicates relative to that in roots. Statistical analyses were undertaken using the Student’s *t*-test. *, *p* < 0.05; **, *p* < 0.01; ns, no significant difference.
