# Supplementary material for: Genome-Wide Identification and Expression Analysis of the Histone Deacetylase Gene Family in Wheat (Triticum aestivum L.)
Source: Plants (Basel). 2020 Dec 24;10(1):19. doi: 10.3390/plants10010019 (PMC7823868; doi:10.3390/plants10010019)
Supplement: Supplementary file 1 [file plants-10-00019-s001.zip › Figure S2.pdf]

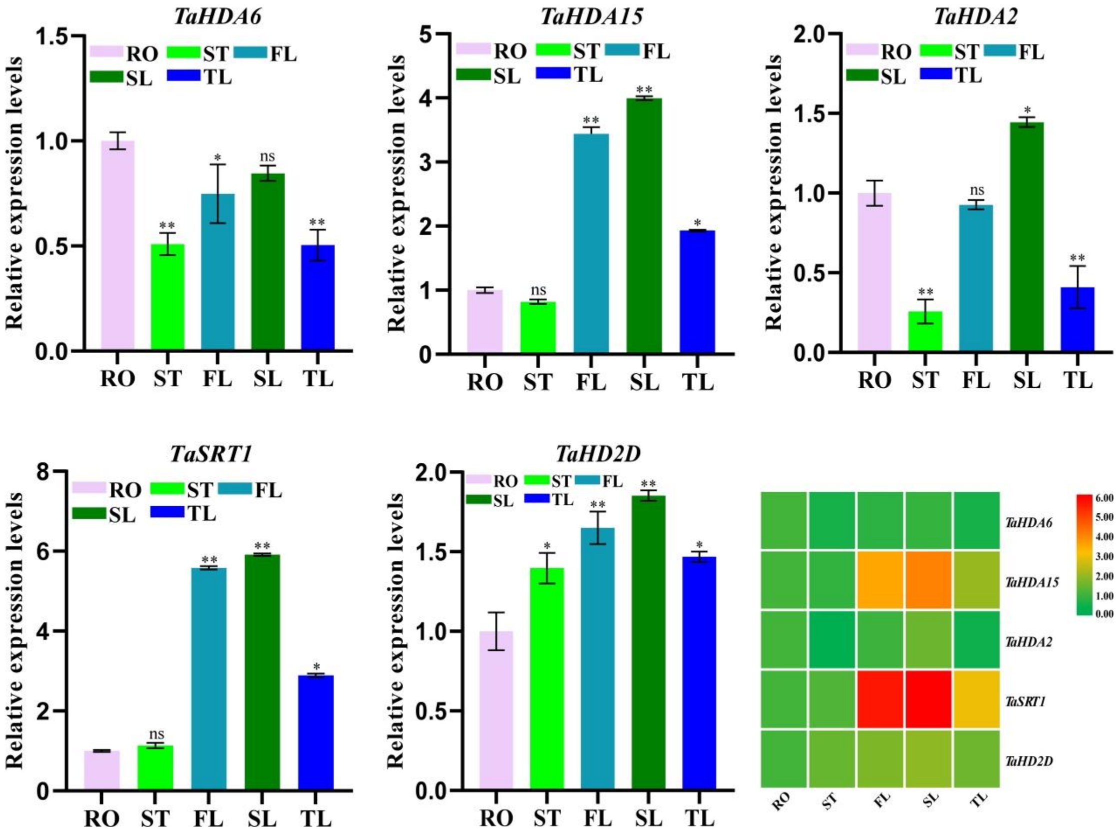

Figure S2. The expression levels of representative *TaHDACs* in diverse tissues by RT-qPCR (raw data). RO: root; ST: stem; FL: first leaf; SL: second leaf; TL: third leaf. The relative expression level of each biological sample was calculated with three biological replicates and three technical replicates relative to that in roots. Statistical analyses were done using the Student's *t*-test. *, $P < 0.05$; **, $P < 0.01$; ns, no significant difference.
