# Supplementary material for: Genome-Wide Identification and Expression Analysis of the Histone Deacetylase Gene Family in Wheat (Triticum aestivum L.)
Source: Plants (Basel). 2020 Dec 24;10(1):19. doi: 10.3390/plants10010019 (PMC7823868; doi:10.3390/plants10010019)
Supplement: Supplementary file 1 [file Figure S1.pdf › Figure S1.pdf]

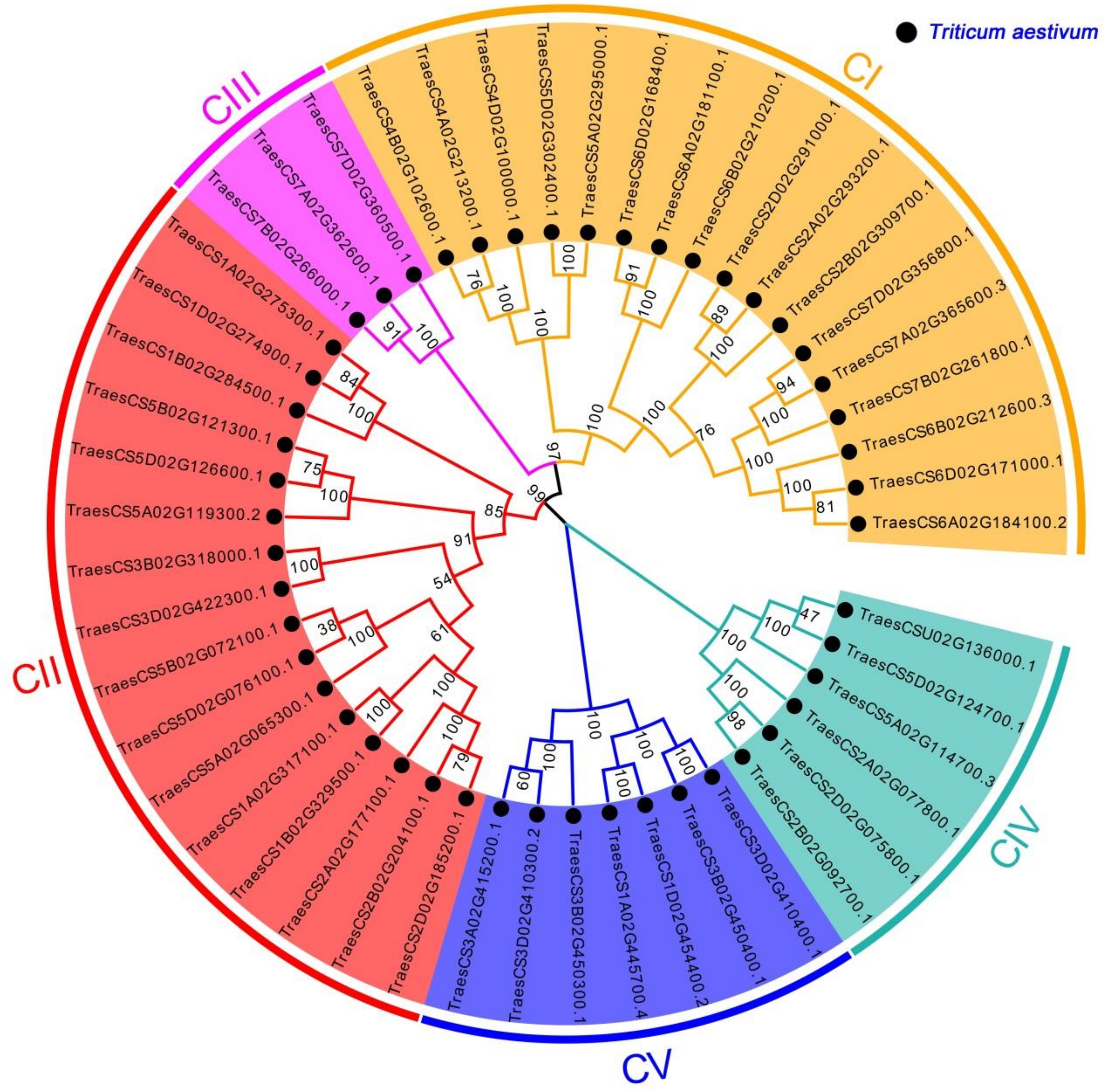

Figure S1. An individual phylogenetic tree was constructed with 49 TaHDACs. All TaHDACs were divided into 5 clades. Orange, red, purple, cyan, and blue represented C I, C II, C III, C IV, and C V, respectively. The unrooted tree was established by the NJ method using MEGA7.0 software with 1000 bootstrap replicates.
